# Genome-Wide Identification of WRKY Family Genes and the Expression Profiles in Response to Nitrogen Deficiency in Poplar

**DOI:** 10.3390/genes13122324

**Published:** 2022-12-10

**Authors:** Yao Chen, Xiangge Kong, Le Yang, Mingyue Fu, Sheng Zhang

**Affiliations:** Key Laboratory of Bio-Resource and Eco-Environment of Ministry of Education, College of Life Sciences, Sichuan University, Chengdu 610065, China

**Keywords:** WRKY transcription factors, *Populus*, nitrogen response, expression pattern

## Abstract

The fast-growing arbor poplar is widely distributed across the world and is susceptible to nitrogen availability. The WRKY transcription factor is an important regulatory node of stress tolerance as well as nutrient utilization. However, the potential response mechanism of *WRKY* genes toward nitrogen is poorly understood. Therefore, the identification of *WRKY* genes on the *Populus trichocarpa* genome was performed, and 98 *PtWRKYs* (i.e., *PtWRKY1* to *PtWRKY98*) were identified. Phylogenetic analysis and the promoter cis-acting element detection revealed that PtWRKYs have multiple functions, including phosphorus and nitrogen homeostasis. By constructing multilayer-hierarchical gene regulatory networks (ML-hGRNs), it was predicted that many WRKY transcription factors were involved in the nitrogen response, such as PtWRKY33 and PtWRKY95. They mainly regulated the expression of primary nitrogen-responsive genes (NRGs), such as *PtNRT2.5A*, *PtNR2* and *PtGLT2*. The integrative analysis of transcriptome and RT-qPCR results show that the expression levels of 6 and 15 *PtWRKYs* were regulated by nitrogen availability in roots and leaves, respectively, and those were also found in ML-hGRN. Our study demonstrates that PtWRKYs respond to nitrogen by regulating NRGs, which enriches the nitrate-responsive transcription factor network and helps to uncover the hub of nitrate and its related signaling regulation.

## 1. Introduction

Transcription factors (TFs) are important constituents of plant signaling pathways that modulate both external stimuli and internal signaling [1]. WRKY TFs are one of the largest plant protein superfamilies and contribute to various processes, including basal immunity, abiotic stress tolerance, nutrient deprivation, seed development, senescence and other developmental and hormone-regulated processes [2,3,4]. These TFs derive their nomenclature from a signature DNA-binding sequence containing the conservative WRKYGQK motif known as the WRKY domain (WD) [5]. They can specifically recognize and bind to a TTGAC(C/T) sequence (W-box) in the promoter regions of downstream target genes [6,7]. The conserved GAC core of the W-box interacts with an α-helix or a β-sheet of the WD, and the flanking thymine and pyrimidine (C/T) residues are recognized by specific WRKY factors [8,9]. In fact, W-box elements are present in numerous genes, including WRKY TFs [10,11,12]. Therefore, WRKY TFs are involved in various pathways and possess auto- and cross- regulation [11,12]. 

WRKY TFs can directly bind with and activate or repress genes encoding transporters, enzymes, structural proteins, and other TFs. In *Arabidopsis*, AtWRKY46 is directly bound to the malate transporter gene *AMLT1* promoter and repressed its expression, leading to reduced malate secretion and increased aluminum accumulation in root apices [13]. Two rice phosphate homeostasis regulation factors, OsWRKY21 and OsWRKY108, regulate the phosphate transporter *OsPHT1;1* directly [14]. Moreover, AtWRKY2 and AtWRKY34 regulated *MYB94* to influence pollen development in *Arabidopsis* [15]. A growing body of research suggests that WRKY TFs regulate several seemingly disparate processes through interactions with other factors [16,17,18]. For instance, binding to the *EDS1* promoter, AtWRKY40 triggered the immune response toward *Pseudomonas syrinyae* in *Arabidopsis* [19]. In addition, AtWRKY40 forms a homodimer negatively regulating ABA response gene *ABI5*, but positively regulates AtWRKY60 when it forms a heterodimer with AtWRKY18 [20,21,22]. Furthermore, associated with MPK3, AtWRKY40 mediates the response of *Arabidopsis* to osmotic and cold stress [23]. In addition to regulating other genes, WRKY TFs are also regulated by many factors. OsWRKY94 is directly activated by OsMADS57 for the cold stress response [24]. RPD3-like histone deacetylase HDA9 inhibits AtWRKY53 to repress the plant response to stress [25]. AtWRKY33 binds to varied proteins to obtain different transcriptional regulatory activities, which forms an inactive complex with MPK1-MKS1 and a strongly activated transcriptional complex with SIB1 and SIB2 [26,27]. The involvement of WRKY TFs in the various processes may be attributed to the complex upstream networks and numerous downstream regulatory targets.

Nitrogen is an essential nutrient and one of the integral factors limiting plant productivity [28,29]. The mechanism of nitrogen absorption and utilization in plants has received extensive attention. As the main form of nitrogen absorbed by land plants, nitrate was transported mainly through several transmembrane transporter families, i.e., NPFs/NRT1s, NRT2s and NRT3s [30,31]. The transcriptional expression level of *NRTs* is always regulated by nitrate availability [32]. This process requires the involvement of multiple TFs [33]. Nitrate is indirectly involved in the synthesis of organic compounds. It should be first reduced to ammonium through two steps and then assimilated into amides and amino acids [33,34]. Therefore, nitrate reduction and assimilation are also pivotal processes affecting plant nitrogen use efficiency (NUE), which are regulated by complex signaling pathways. Enzymes (such as nitrate reductase and nitrite reductase) mediating nitrate reduction are induced at the transcriptional and protein levels [34]. Glutamate reductase, which further mediates the assimilation of ammonia into amino acids, is also strictly regulated [34]. All these processes are inseparable from the participation of transcription factors. Therefore, investigating TFs involved in nitrogen utilization is beneficial to unravel the nitrogen regulation networks. 

Plants sense nitrate signals through phosphorylation of NRT1.1 and transduce them into the nucleus mainly through the involvement of TFs, leading to instantaneous or systemic responses [35,36,37]. The first TF identified in the nitrogen response was the MADS-box gene *ANR1* [38]. Later, SPL9 was predicted to be a potential nitrogen response hub [39]. AtNLP7 is regarded as the master regulator of the primary nitrogen response regulating other TFs to broadly impact nitrate signaling [40]. Recently, more nitrogen response TFs were reported, such as the basic helix-loop-helix TF HBI1 [41], the light response TF HY5 [42] and the Dof TF OBP4 [43]. Currently, via gene expression analysis, some studies have confirmed that WRKY transcription factors are involved in the nitrogen response process of plants. Early in the analysis of transcriptome data of *Arabidopsis* roots after nitrate treatment, many differentially expressed WRKY genes were identified [44]. Gene expression analysis after nitrogen treatment in *Withania somnifera* detected an increase in *WsWRKY1* expression levels [45]. In *Arabidopsis*, nitrogen-responsive gene regulatory network (GRN) analysis and gene function defect analysis implicated that AtWRKY1 mediated crosstalk between light and nitrogen signaling pathways [2]. In addition, stevia treated with different forms of nitrogen was sent for transcriptome sequence, and there were five differentially expressed WRKY genes found among the 23 differentially expressed transcription factors identified, occupying a large proportion [46]. Although WRKY transcription factors have long been involved in the plant nitrogen response, the specific roles remain unclear. 

Poplar is often used as a pioneer tree for artificial afforestation due to its fast growth and easy reproduction, and it mostly lives in nitrogen-poor soils [47,48,49]. Poplar has finer wood properties and stronger resistance to disease or abiotic stresses with sufficient nitrogen use [50,51,52]. However, little is known about the molecular regulatory network and the role of WRKY TFs in responding to fluctuating nitrogen availability in poplar. In this study, we used the latest genomic data information to reidentify the poplar WRKY transcription factor family, combined with the existing research and annotation results of multiple databases to analyze and predict the sequence information and biological functions of all members. WRKY transcription factors with nitrogen-responsive functions were analyzed and screened, and their response mechanisms were predicted by constructing a gene regulatory network. It was verified by RT-qPCR. Here, we used a bioinformatic approach coupled with genetic network analysis to study the responses of the poplar root system to nitrogen deficiency. We show the mechanism by which WRKY transcription factors are involved in the crosstalk processes that regulate nitrogen responses and other signals in poplar, providing a theoretical basis for the genetic breeding of poplars with higher NUE.

## 2. Materials and Methods

### 2.1. Plant Materials, Growth Conditions and Nitrate Deficiency Treatments

*Populus cathayana* Rehd. is a member of Sect. *Tacamahaca Spach* and has been reported to be frequently affected by nitrogen deficiency [49]. 84K poplar (*Populus alba×Populus glandulosa*) is a hybrid specie of Sect. *Leuce Duby*, a widely planted tree and a good genetic transduction material [53]. Here, *P. cathayana* was collected from Qinghai, China, and 84K poplar was preserved in the greenhouse of Key Laboratory of Bio-Resource and Eco-Environment of Ministry of Education, Sichuan University. Poplar seedlings were grown in WPM medium in an incubator (25 °C constant temperature, 16 h/8 h light–dark cycle, 10,000 Lux light intensity, and 65% relative humidity). For nitrate deficiency treatments, one-month-old plants with similar growing conditions and identical genetic backgrounds were selected. Modified Hoagland solution was used to culture the poplars in the control group (CK) and without nitrate in the nitrate-deficient treatment group (ND) [49]. Each group had at least 20 replicates. Samples were collected at 3:00 pm after three weeks of treatment. All samples were stored at −80 °C after freezing in liquid nitrogen for subsequent quantitative real-time PCR analysis.

### 2.2. Identification and Physicochemical Properties Analysis of PtWRKYs

As the first poplar to have its genome sequenced, *P. trichocarpa* has abundant gene and protein annotations. The HMM profile of the WRKY DNA-binding domain (PF03106), which was downloaded from the Pfam database (http://pfam.sanger. ac.uk/, accessed on 25 August 2022), was used to identify the WRKY gene from the genome of *P. trichocarpa* via hmmsearch with default parameters (e-value cut off <10^−5^) [54]. Based on *P. trichocarpa* genome sequencing data (https://phytozome-next.jgi.doe.gov/info/Ptrichocarpa_v4_1, accessed on 25 August 2022), all putative WRKY proteins of *P. thrichocarpa* were retrieved by searching against the transcriptome annotation file with the keyword “WRKY”. Simultaneously, all WRKY TFs of *P. trichocarpa* in the PlantTFDB database (http://planttfdb.gao-lab.org/, accessed on 25 August 2022) were downloaded. The results obtained using the three methods together with previous studies were integrated and rechecked to avoid repetition [55,56]. The WRKY protein sequences of *P. trichocarpa* were downloaded to search the WRKY domain using the SMART program (http://smart.embl-heidelberg.de/, accessed on 3 September 2022), and multiple alignment was conducted against *Arabidopsis thaliana and Oryza sativa* WRKY protein sequences using the Clustal X tool (Version 1.83, Higgins et al., Dublin, Ireland) to remove repetitions and sequences with incomplete WRKY domains. The putative WRKY genes from *P. trichocarpa* were named based on previous studies and AtWRKY homologs.

The basic physicochemical properties of PtWRKYs were analyzed by ProtParam (https://web.expasy.org/protparam/, accessed on 3 September 2022), including the theoretical isoelectric point (pI), molecular weight and hydrophilicity. The online software WOLF PSORT (https://wolfpsort.hgc.jp/, accessed on 3 September 2022, Horton et al., Tokyo, Japan) was used to predict the subcellular localization of poplar WRKY TFs. 

### 2.3. Chromosome Map and Collinearity Analysis of PtWRKYs

The gene position information of 98 identified *PtWRKYs* was downloaded from the genome database Phytozome V13 (https://phytozome-next.jgi.doe.gov/, accessed on 3 September 2022), and the chromosome length information of *P. trichocarpa* was downloaded from NCBI. The chromosome distribution map was drawn using the online toolkit MG2C (Version 2.1, http://mg2c.iask.in/mg2c_v2.1/, accessed on 3 September 2022). 

The multiple collinearity toolkit MCScanX was used to analyze the gene duplication events of poplar WRKY transcription factor family members [57]. Collinearity analysis was performed between poplar WRKY transcription factor family members and other species including *Arabidopsis*, rice, apple and willow. Collinear graphs were drawn using the Circos tool and multiple synteny plot tool in TBtools (Version 1.098769, Chen C.J., Guangzhou, China) [58].

### 2.4. Protein Sequence Alignment and Phylogenetic Tree Construction

The protein sequences of all poplar and *Arabidopsis* WRKY TFs were downloaded from the genome database Phytozome V13 (https://phytozome-next.jgi.doe.gov/, accessed on 3 September 2022). Multiple sequence alignment of these WRKY proteins was performed using the software Clustal X. The alignment results were imported into Jalview (Version 1.18, Clamp et al., Yokohama, Japan) to visualize the WD sequence characteristics of each group and then imported into MEGA7 (Version 7.0, Kumar et al., Philadelphia, USA) for phylogenetic tree construction using the neighbor-joining method [59]. The bootstrap value was set to 500, and other parameters were default. The calculated results were visualized and further optimized using the online tool Evolview (Version 3.0, Subramanian et al., Wuhan, China) [60].

### 2.5. Motif and Sequence Structure Analysis of PtWRKYs

The protein sequence file of PtWRKYs was uploaded to the MEME toolkit (Version 5.4.1, Timothy et al., San Diego, USA) [61], the number of motifs to be searched was set to 6, and the remaining parameters were default. Gene position information was downloaded. Gene names, motif analysis results, and gene structure annotation information of PtWRKYs were simultaneously imported into the Gene Structure View tool of TBtools for visualization.

### 2.6. Functional Annotation of PtWRKYs

Many methods have been developed to identify the function of proteins. Phylogenetic analysis is one of the most rapid and relatively accurate approaches to identify the function of TFs according to the conservation of protein sequences. Literature analysis and phylogenetic tree construction were integrated to predict the biological functions of PtWRKYs. Studies containing AtWRKYs, OsWRKYs or PtWRKYs were searched, the known functions of these WRKYs were summarized and listed, and the abundance of WRKYs under each function directory was counted. The phylogenetic tree was constructed to analyze the genetic relationship of WRKY proteins in the three species. The function of PtWRKYs was annotated according to the WRKYs with the highest homology of known functions. The prediction results were compared with some previous reports to verify the accuracy.

### 2.7. Construction of a Multilayered Hierarchical Gene Regulatory Network Using a Recursive Random Forest Algorithm

A random forest-based algorithm, namely, backward elimination random forest (BWERF), was used for the construction of the TF-based ML-hGRN response to nitrogen [62]. Briefly, a pathway gene expression matrix and a TF expression matrix were input. For each pathway gene, a backward elimination random forest model was constructed recursively. Aggregation of the importance values of a TF to all pathway genes was performed to produce a unified the importance value of this TF to the pathway. The most important TFs were identified and used as a layer. Using the new TF layer as the bottom layer, all the above-mentioned procedures were repeated to obtain the next layer until the designated number of layers was achieved or the program was terminated due to the lack of significant TFs as input for the upper layers. In this study, a nitrogen response gene expression matrix with 26 items was prepared (Supplementary Table S1). Simultaneously, a gene expression matrix containing all transcription factors and another containing only WRKY transcription factors were prepared (Supplementary Table S1). Next, the BWERF program was run to calculate the interfering effects of all WRKY transcription factors and WRKY transcription factors on NRG separately. The TF layer was set to 3 and the elimination ratio was 1/10. Finally, the results calculated by the BWERF algorithm were visualized using Cytoscape (Version 3.9.1, Shannon et al., Seattle, USA) [63].

### 2.8. RNA Isolation, Reverse Transcription and RT-qPCR Detection

Total RNA was extracted from different nitrate-treated leaf and root samples using the RNAprep Pure Plant Plus Kit (DP441; TIANGEN Biotech Co.,Ltd., Beijing, China) following the manufacturer’s instructions. Total RNA (1 μg) was treated with 10 U DNase I to remove residual genomic DNA and then used for reverse transcription with 2× Hifair II SuperMix (11120-B; Yeasen Biotechology Co.,Ltd, Shanghai, China). The expression levels of WRKYs from nitrate deficiency treatment were detected by quantitative real-time polymerase chain reaction (RT-qPCR), and the 18S rRNA coding gene was selected as the reference gene. Fifteen WRKYs were randomly selected to verify the expression profile of WRKYs under nitrate-deficiency treatment, and the specific primers were designed by Primer 6 software and BLAST in NCBI. RT-qPCR was performed on a CFX Connect platform (Bio-Rad, Hercules, CA, USA) with the fluorescent reagent Hieff® qPCR SYBR Green Master Mix (11201ES03; Yeasen Biotechnology Co., Ltd., Shanghai, China). The following qPCR conditions recommended by the manufacturer were used: 95 °C for 2 min, 40 cycles at 95 °C for 10 s, and 60 °C for 5 s. The experiments were repeated three times, and ultrapure water was selected as a negative control. WRKY gene expression levels were calculated using the 2^−ΔΔCt^ method [64]. RT-qPCR data were technically replicated, with error bars representing the mean ± SD (*n* = 3).

### 2.9. Statistical Analysis

The gene sequences, annotation and expression of all species mentioned were derived from the databases Phytozome v13 (https://phytozome-next.jgi.doe.gov/, accessed on 3 September 2022), and TAIR (https://www.arabidopsis.org/, accessed on 3 September 2022). Data were analyzed by Excel for regular calculations, and SPSS (Version 19.0, IBM Corp., Armonk, NY, USA) was used for ANOVA, Turkey test and Student’s t test (*p* ≤ 0.05). Plots were drawn with GraphPad Prism (Version 8.0.2, GraphPad Software, LLC., San Diego, CA, USA).

## 3. Results

### 3.1. Identification of WRKY Family Members in P. trichocarpa

A total of 98 *PtWRKY*s were characterized from the whole genome, and the ID and names of the obtained *PtWRKYs* were listed in Supplementary Table S2. The multiple alignment results reveal that all 98 PtWRKYs contained WRKY domains, but a difference was found in the WRKYGQK motif. Further analysis indicated that 92 PtWRKYs contained conserved WRKY domains (WRKYGQK), PtWRKY34 comprised a FRKYGQK mutation, PtWRKY26/63 comprised a WKKYGQK mutation and PtWRKY50/51/59/97 comprised a WRKYGKK mutation (Supplementary Figure S1). The 98 PtWRKY proteins were classified by the method of Thomas and divided into three groups (groups I, II, and III) plus five subgroups in group II [5]. As previously described, there were 21 WRKY proteins in group I that had two WRKY domains (WD) with a C2H2-type zinc-finger. There were also 10 WRKY proteins in group III having one WD with a C2HC type zinc finger. In addition, the remaining 67 WRKY proteins having one WD with a C2H2 type zinc figure belonged to group II and were divided into five subgroups (IIa, IIb, IIc, IId and IIe), including 5, 9, 24, 13 and 16 proteins, respectively (Supplementary Figure S1 and Table S2).

### 3.2. Physicochemical Property, Motif and Gene Structure of PtWRKYs

Based on the protein sequence of PtWRKYs, the physicochemical properties were predicted and analyzed. Since group I WRKY proteins contain two WDs, they had significantly longer amino acid (aa) sequence lengths and larger molecular weights than the other groups. In detail, the group I members were 368-739 aa in length (with an average of 549 aa) and 40.9–79.6 kD in weight (with an average of 60.15 kD). In addition, other group members had an average length of 331 aa (from 135 to 631aa) and an average molecular weight of 36.7 kD (from 15.5 to 67.9 kD). Furthermore, the sequence length and molecular weight of the subgroup IIc proteins were relatively larger, second only to group I. The theoretical isoelectric point (IP) of the PtWRKY protein was between 5.08 and 9.86. The composition was as follows: 50%—acidic proteins, 44.9%—basic proteins, and the remainder were nearly neutral (Supplementary Table S2). All PtWRKYs were subcellular localized in the nucleus, consistent with their transcription factor characteristics.

**Figure 1:**
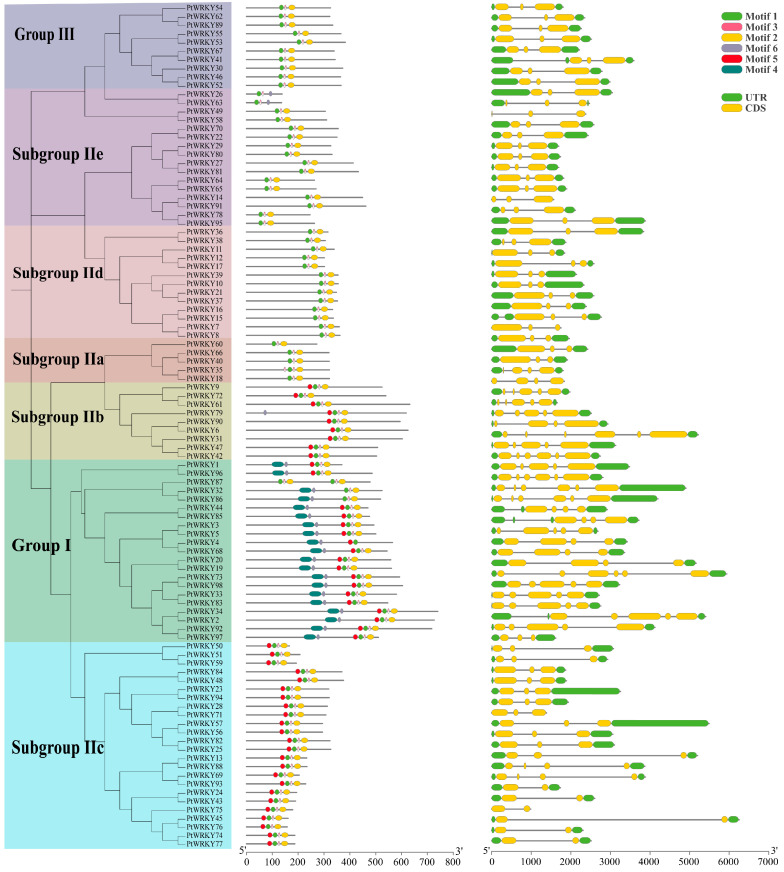
Motifs and gene structure of poplar WRKY transcription factors. From left to right, the phylogenetic tree, motif distribution and gene structure of WRKY transcription factors are shown. Different subgroups of PtWRKYs were distinguished by different color backgrounds.

The MEME program was used to predict the conserved motifs of PtWRKY proteins, and six conserved motifs identified (namely, motifs 1 to 6) were all distributed in the WRKY domain of each protein. The obtained results show that motif 4 was found only in group I, and motif 1 was also mainly found in group I, and both together formed its N-terminus WRKY domain. The WRKY structure at the C-terminal of group I contained four other motifs, and the motif composition was the same as that of the WRKY domain of subgroups IIb and IIc. Group II and III proteins lacked motif 5 at the N-terminal compared with the C-terminus WRKY domain of group I (Figure 1).

To illuminate the evolutionary process, the number and arrangement of introns exons of 98 *PtWRKYs* were analyzed. The obtained results show that genes in the same subgroup had similar intron–exon structures. Most *PtWRKYs* contained two introns, and a few contained 1, 3, 4 or 5 introns. The characteristics of group I members were more obvious, all containing 3–5 introns, and the number was significantly greater than that of other subgroups. The subgroup IIc proteins contained only two introns and their arrangement characteristics were similar to those of the 3’ end of the group I, with long intron insertions. Subgroup IIb genes contained four to five introns and were arranged similarly to group I members. The numbers and distribution of introns in group III, subgroups IId and IIe were similar, and generally included two insertions of close length (Figure 1).

### 3.3. Chromosome Mapping and Gene Duplication Events

**Figure 2:**
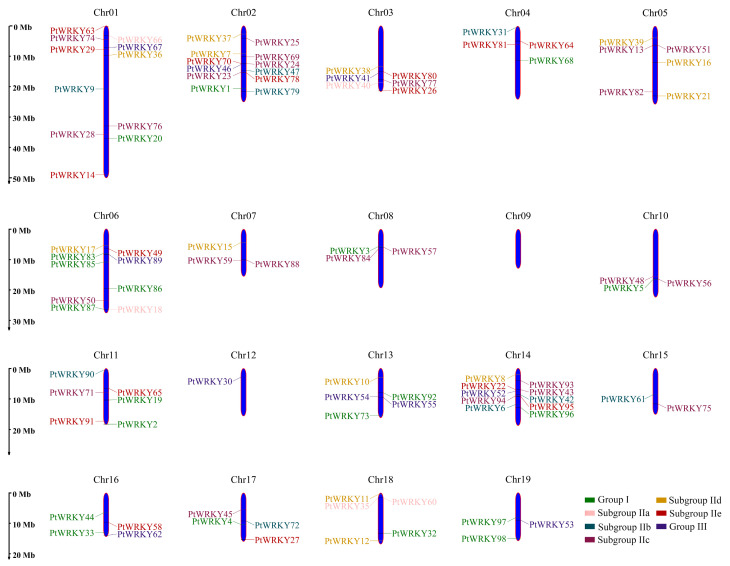
Chromosomal mapping of *PtWRKYs*. The blue bars displayed the chromosome of poplar. Genes IDs with different colors belong to different subgroups.

Fragment duplication and tandem duplication are the main causes of plant gene family expansion, and gene sequences derived from the expansion of the same locus often have high similarity. Based on the genome annotation information, the chromosome map of *PtWRKYs* was drawn, and all 98 *PtWRKYs* had their specific locations (Figure 2). They are irregularly and unevenly distributed on all chromosomes except chromosome 9. The distribution of *PtWRKYs* was relatively concentrated on chromosomes 1, 2, 6 and 14; only *PtWRKY30* was distributed on chromosome 12; only *PtWRKY61 and PtWRKY75* were distributed on chromosome 15; and 3~6 *PtWRKYs* were distributed on the other chromosomes. At the same time, it was found that subgroup IIa genes were all distributed at the end of the chromosome, and the other groups were distributed at any position of the chromosome. The wide distribution of *PtWRKYs* in the genetic ancestor suggests that *PtWRKYs* may play an important role in the complex life process of poplar.

**Figure 3:**
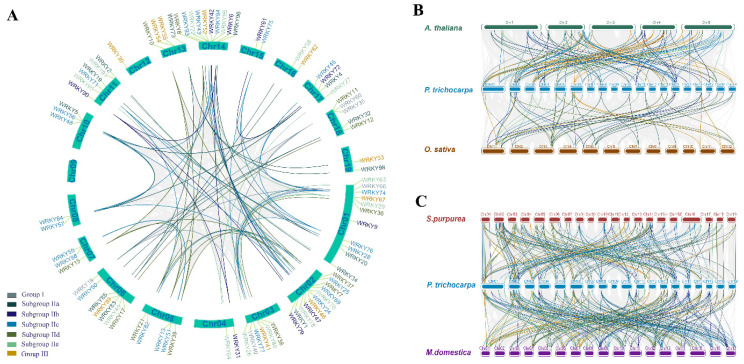
Intraspecific and interspecific collinearity analysis of *WRKYs*. Intraspecific collinearity of PtWRKYs (**A**). Interspecific collinearity analysis of PtWRKYs against *Arabidopsis* and rice (**B**), or against willow and apple (**C**).

To unravel the gene replication and expansion events of *PtWRKYs*, collinearity analysis was conducted. The obtained results show that there was a wide range of collinearity in the WRKY gene family in poplar. Most of linearly related genes were located on different chromosomes. All *PtWRKYs* on chromosome 3 are linearly related to some of those on chromosome 1, and some *PtWRKYs* on chromosome 2 are closely related to that in chromosome 14 (Figure 3A). Analysis against *WRKYs* in other plants indicated that *PtWRKYs* showed a strong collinearity related to most *WRKYs* in *Arabidopsis*, but little collinearity in *Oryza* (Figure 3B). Thus, the function of *PtWRKYs* will be further predicted by homologous alignment carried out with *AtWRKYs*. In addition, *PtWRKYs* were strongly correlated with *WRKYs* in *Salix purpurea* and *Malus domestica* (Figure 3C). 

### 3.4. Cis-Acting Element Analysis of PtWRKY Promoters

**Figure 4:**
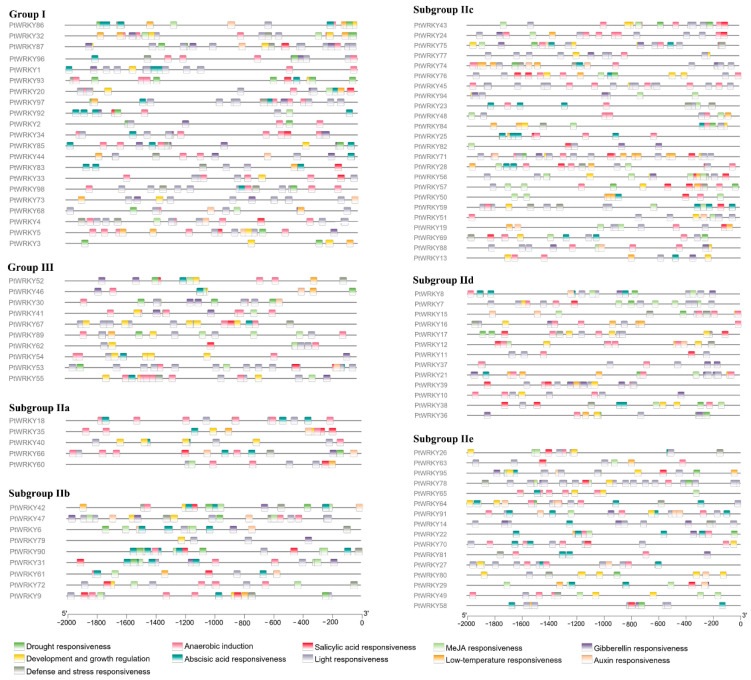
Cis-acting elements in *PtWRKYs* promoter. Line represents 2000 bp gene upstream sequence, and different color rectangles represent different response elements.

Screening in the promoter region of *PtWRKYs* revealed these main cis-acting elements (Figure 4). *WRKYs* are always widely involved in the crosstalk of various signals, mediating the regulation of almost all life activities, such as plant growth, development, and stress response. Among these cis-acting elements, light-responsive elements were the most numerous, and except for subgroup IId, which contains only a few ABA-responsive elements, the rest of the subgroup members contain a large number of ABA-responsive elements. This result suggested that *PtWRKYs* were extensively involved in the regulation of poplar photomorphogenesis and ABA signaling pathways. There was no apparent preference for these cis-acting elements between different subfamilies, and even the promoter cis-acting elements of members with extremely high homology are not similar, which can make proteins with the same (or similar) functions affected by upstream regulation of different signals perform different functions.

### 3.5. Functional Annotation of PtWRKYs

**Figure 5:**
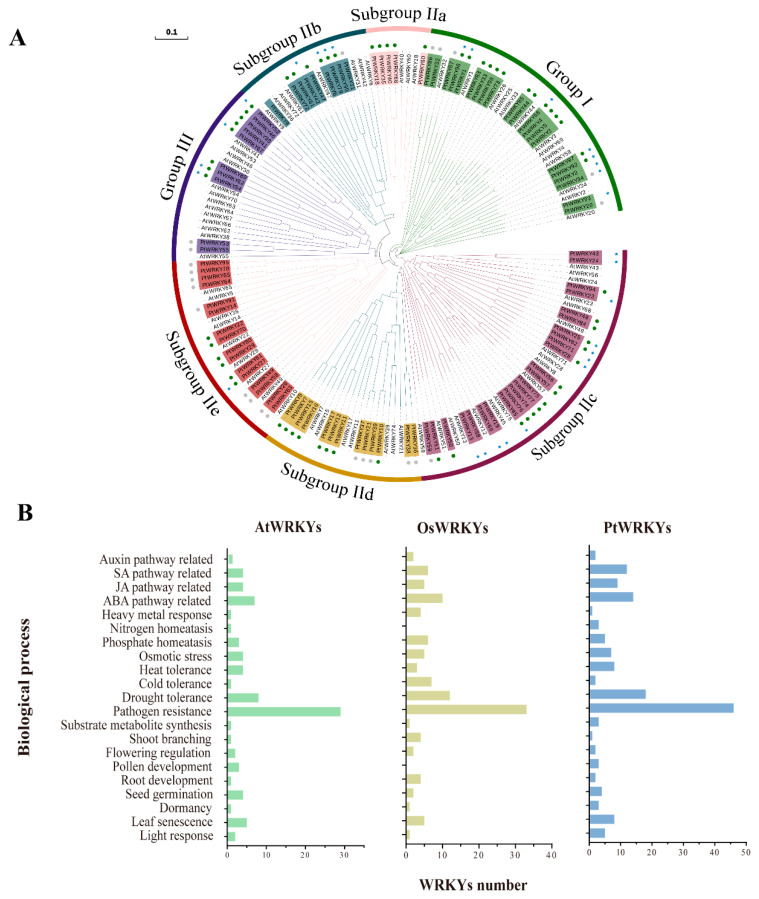
Functional annotations of WRKY TFs. Polygenetic tree WRKY TFs in poplar and *Arabidopsis* (**A**). Different colors were used to mark the phylogenetic branches of WRKY transcription factors in different subgroups. The protein names of the different subgroups of PtWRKYs are highlighted using a background of different colors, and AtWRKYs is not highlighted. Circles and stars following the protein name indicate the predicted physiological function of the protein, respectively: green circles indicate stress response function, gray circles indicate unknown function, and blue stars indicate growth and development regulation function. Previous reported functions of WRKY TFs in *Arabidopsis* and rice and putative functions of WRKY TFs in poplar (**B**).

Biological function analysis has always been the core area of research in TFs. It is clear and superficial that GO annotations of WRKY transcription factors in databases are always limited to their transcriptional regulatory functions. The phylogenetic tree clustered the WRKY transcription factor subfamilies together, which could be intuitively divided into seven subgroups (Figure 5A). The numbers of PtWRKYs in each group were 21 (group I), 5 (subgroup IIa), 9 (subgroup IIb), 24 (subgroup IIc), 13 (subgroup IId), 16 (subgroup IIe) and 10 (group III). Among them, the members of subgroup I are genetically distant from the other subgroups due to the two WRKY domains, and the genetic distance within the group is relatively distant. The members of the subgroups IIa, IIb, IIc and IId were genetically closer to those of the group III, and they may have gradually evolved and enriched with the complexity of plant functions.

We made bold predictions of the functions of PtWRKYs on the basis of the known functions of AtWRKYs and OsWRKYs (Supplementary Table S3). Studies have mainly focused on the regulatory role of WRKY proteins in plant stress responses, especially in disease resistance and basic immune regulation (Figure 5B). More than half of WRKY TFs in both *Arabidopsis* and rice have been confirmed to be involved in the biological stress response, possibly by mediating JA and SA signaling pathways or directly regulating the transcription of *R* genes. In addition, the second most focused function of WRKYs is related to drought resistance. PtWRKYs involved in other abiotic stress response processes were also abundant, including high temperature, low temperature, osmotic stress, and heavy metal stress. In addition, few WRKYs were reported to be involved in phosphorus and nitrogen homeostasis, such as AtWRKY1 (Supplementary Table S3).

### 3.6. Construction of ML-hGRNs That Regulate the Poplar Nitrogen Response

**Figure 6:**
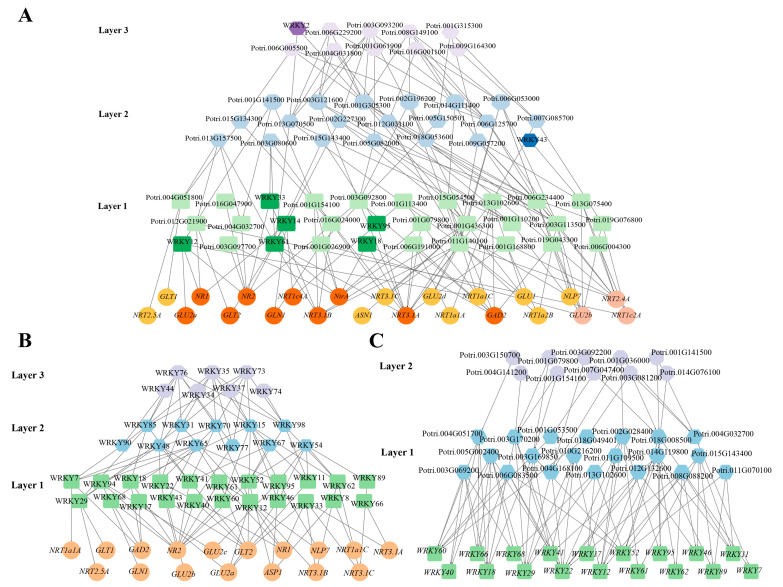
PtWRKYs involved in nitrogen response. Gene network of PtWRKYs regulating NRGs. Gene regulatory network of PtWRKYs response to nitrate. Different layers were distinguished by different colors. The WRKY TFs and the genes it directly regulated were highlighted in darker colors.

We applied the BWERF algorithm to the nitrogen response pathway for reverse-engineering ML-hGRNs. The last layer was considered to govern the nitrogen primary response, including genes involved in nitrate transport, reduction and assimilation (Figure 6). The ML-hGRNs constructed from NRGs and all TFs in poplar indicated that PtWRKYs played a role in the nitrogen response. The obtained results show that there were eight PtWRKYs involved in the regulation of NRG expression, accounting for 13% of all transcription factors in the network (Figure 6A). Six PtWRKYs were shown in the first layer. That is, they probably regulate NRGs directly. To screen for more potential nitrogen response-related PtWRKYs, an NRG-based ML-hGRN was further constructed with only PtWRKYs (Figure 6B). Clearly, in addition to the above-mentioned eight PtWRKYs, others (such as PtWRKY40, PtWRKY60 and PtWRKY22) were also potential regulators of NRGs. These PtWRKYs were also regulated by many other transcription factors at the transcriptional level (Figure 6C).

### 3.7. Expression Analysis of PtWRKY Genes under Nitrogen Treatment

**Figure 7:**
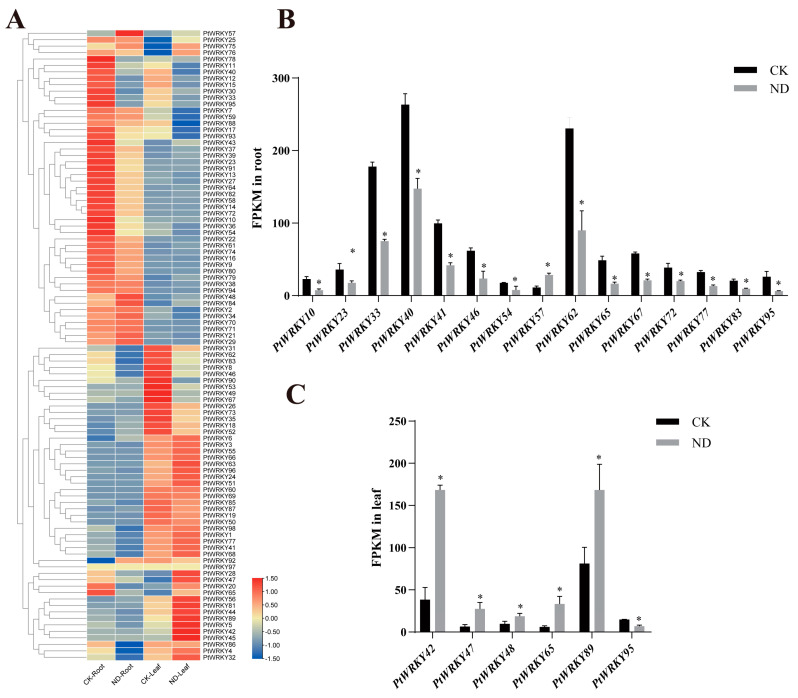
*PtWRKYs* transcription expression level under LN. Heat map of *PtWRKYs* expression obtained from transcriptome sequence data (**A**). Differently expressed *PtWRKYs* in poplar roots (**B**) or leaves (**C**). CK, the control group; ND, the nitrogen deficiency group. According to independent sample *t*-test results, “*” indicated significant difference in gene expression compared with control (CK) (*p* ≤ 0.05).

Our previous nitrogen-deficient transcriptome sequencing data of poplar showed that in poplar seedlings treated with nitrogen deficiency for two months, the expression levels of some WRKY genes changed significantly in both roots and leaves compared with the control, but the trend was different (Figure 7A). There was a total of 19 *PtWRKYs* with significantly different expression levels. Among them, PtWRKY42, PtWRKY47, and PtWRKY48 were significantly upregulated in leaves, but PtWRKY95 was downregulated (Figure 7B). Moreover, in roots, *PtWRKY40* and 13 other genes were downregulated, and *PtWRKY57*, in contrast, was upregulated (Figure 7C).

**Figure 8:**
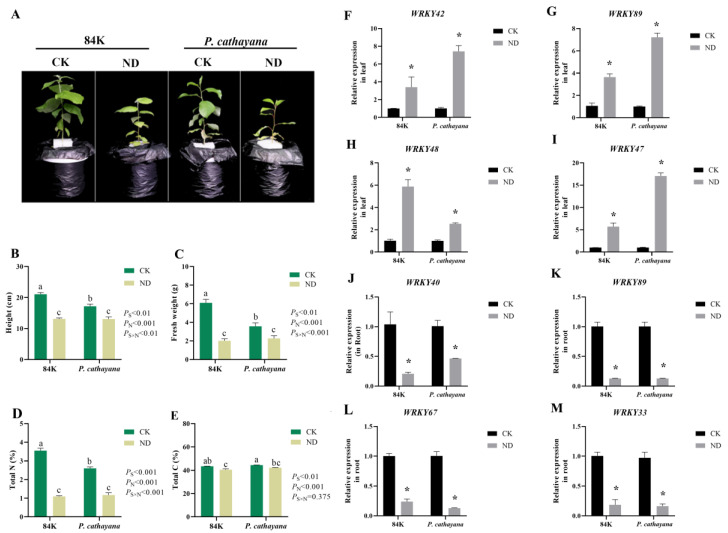
RT-qPCR analysis of PtWRKYs responding to nitrate. Growth inhibition of poplars treated after nitrogen deficiency (**A**–**E**). The relative expression levels of PtWRKYs in different poplar leaves (**F**–**I**) and roots (**J**–**M**) after nitrogen deficiency. CK, control group with normal nitrogen; ND, N-deficiency treatment group. Each set had three biological replicates and showed the mean ± standard error. According to the ANOVA analysis of the Turkey test, different letters indicate significant differences (*p* ≤ 0.05). According to independent sample *t*-test results, “*” indicated significant difference in gene expression compared with control (CK) (*p* ≤ 0.05).

To validate the ML-hGRN prediction and transcriptome sequencing results, we subjected 84K poplar and *P. cathayana* to nitrogen-deficiency treatment and determined the *PtWRKYs* transcriptional expression level by RT-qPCR. Nitrogen deficiency significantly inhibited the growth of poplar and changed its carbon and nitrogen balance (Figure 8A–E). Four differentially expressed *PtWRKYs* in roots and leaves were randomly selected for RT-qPCR analysis, and the results show that their expression changes were basically consistent with the transcriptome sequencing results, and the changes were consistent among different species (Figure 8F–M).

## 4. Discussion

WRKY TFs are one of the largest superfamilies because of their expansion caused by successive duplication events in many species during evolution [9,65]. It is not difficult to identify WRKY TFs because they have one and only one characteristic functional domain, and their structural and functional features are very distinct. Since the first discovery of WRKY TFs in 1994 [66], a large number of WRKY TFs have been identified in different species including poplar [55,56]. WRKY TFs seem to be more abundant in species with more complex genomes. As shown in studies, there are 75 WRKYs in *Arabidopsis* [67], 126 in *O. sativa* ssp. *japonica* [68], 81 in *Solanum lycopersicum* [69], 48 in *Camellia japonica* [70], and 197 members in *Glycine max* [71]. Poplar, as a woody plant, has a large genome and contains a large number of WRKYs. This has led to a slight controversy about the composition of poplar WRKYs. Different studies and databases have different annotations for PtWRKYs. In the present study, a total of 162 transcripts above the threshold were screened by hmmsearch. After removing the duplicates due to variable clipping and the members with incomplete WD, only 98 members remained (Supplementary Table S2), accounting for approximately 0.28% of the total proteins. Unlike the results of He et al. [55], the number of PtWRKYs was reduced by 24 compared to 122. In addition, five redundant items in the TFDB database and two incorrect annotation items in the *Phytozome* database were removed. Due to the complex membership of PtWRKYs, the early naming system is confusing, which is detrimental to long-term studies. Therefore, based on the names of some PtWRKYs with known functions, this study named them after the names of AtWRKY homologs, which is helpful to standardize the PtWRKY research system.

According to the structure of WRKY proteins, the WRKYs of higher plants are primarily divided into three groups and seven subgroups [5]. In the present study, 98 PtWRKYs were grouped into similar categories with 75 AtWRKYs by protein alignment, phylogenetic tree analysis, and conserved motif analysis. PtWRKYs had all subgroups and each subgroup had a specific motif. Another classification method supported by the evolutionary relationship of WDs divided the WRKY TFs into four categories, namely, I, IIa+IIb, IIc, and IId+IIe+III [72]. This finding is consistent with the clustering results of our phylogenetic tree. Meanwhile, the classification results of the present study revealed that the largest number of PtWRKY proteins belonged to subgroup IIc, and the least number of PtWRKY proteins belonged to subgroup IIa. Furthermore, the results of the motif analysis indicate that motifs 1 and 2 were highly conserved in all PtWRKYs, which may correspond to the WDs.

Phylogenetic analysis has become one of the most rapid and relatively accurate approaches used to identify the function of TF proteins according to the conservation of protein sequences [9]. Previous reports on the function of homologous WRKY proteins in other plant species may serve as a reference for verifying the potential roles of PtWRKYs. Several studies have been conducted on various model plants to elucidate the mechanism and function of WRKYs. These studies have particularly focused on PtWRKYs regulating tolerance to biotic and abiotic stresses, including pathogens, wounding, drought, cold, salt, hypoxia, heat and VU-B [11,73,74,75,76]. In addition, WRKY transcription factors are related to plant morphogenesis and metabolite synthesis, as well as important regulators of plant hormone signaling, and play an important role in plant local or systemic stress responses [1]. Therefore, a phylogenetic tree and annotation plots based on previous studies were conducted to determine the functions of PtWRKYs (Figure 5). The functions of PtWRKYs were divided into 21 categories, mainly involved in plant hormone signaling pathways, biotic stress response, abiotic stress responses, nutrient element uptake, plant morphogenesis and so on (Figure 5 and Supplementary Table S3). Clearly, WRKYs with pathogen resistance functions were the most abundant in both *Arabidopsis* and rice, which may be attributed to the stronger preference of researchers for this aspect of WRKY function when studying its function [9]. Therefore, it is possible that these genes also contain other functions. In fact, a large amount of data indicates that a particular WRKY TF is often simultaneously involved in several plant life processes. For example, AtWRKY6 modulated both seed germination and phosphate homeostasis [77,78], PtWRKY75 regulated JA and ROS pathways [79,80], and OsWRKY72 was involved in flowering regulation, stem development and osmotic stress tolerance. Normally, WRKY TFs activate or repress the transcriptional expression of target genes by binding to the widely spread W-box in the promoter region with their WD [80]. This is one of the reasons why WRKY TFs play an important role in signaling within plant cells. In-depth exploration of the functional diversity of WRKYs will help to provide new insights into the functional characteristics of WRKYs, and is more conducive to clarifying the role of WRKYs in the process of multi-signal crosstalk in plant cells, such as nutrients, pathogens, stress and hormones [81,82,83,84]. This finding lays a theoretical foundation for the subsequent functional identification of PtWRKYs.

Nitrogen is not only a key nutrient element, but also an important signaling molecule that regulates morphogenesis, basal immunity and the stress response of plants [84,85]. Plants sense NO_3_^-^ and then transduce it into intracellular signals through complex pathways [33]. A nitrate-CPK-NLP module, mediated by the plasma-nuclear shuttle of AtNLP7, found in *Arabidopsis*, is now a well-recognized mechanism for nitrate signal turning into the nucleus [36]. After entering the nucleus, AtNLP7 is involved in regulating the expression of many nitrogen-responsive genes [36]. However, studies have found many NLP7-independent NRGs, suggesting that there are other undiscovered core factors in the plant nitrogen response regulatory network [39,41,86]. In this study, ML-hGRN was constructed for the regulation of all transcription factors to NRGs in poplar, and multiple PtWRKYs were found, suggesting that PtWRKYs played a role in regulating the nitrogen response (Figure 6A). In addition, other transcription factor families, such as MADS-box, bZIP, and AP2_ERF, were also found in the NRG-based ML-hGRN, which was confirmed in model plants (Figure 6A). For example, ANR1, a transcription factor first identified in the nitrate response, and LBD37, LBD38 and LBD39, which play negative regulatory roles under nitrate starvation, all belong to the MADS-box family [38,87]. Members of the bZIP family involved in the nitrogen response included bZIP1, a regulator responsible for nitrogen and light crosstalk, TGA1 and TGA4, which regulate the occurrence and development of lateral roots in *Arabidopsis*, and NRG2, which regulates NRT1.1 expression [88,89,90]. In addition, the JA-related AP2_ERF transcription factor ERF1 was significantly induced under sufficient nitrogen, which made plants exhibit higher disease resistance under high nitrogen conditions [91]. 

Bioinformatics is an important method to predict the function of unknown proteins in modern molecular biology [92]. In this study, the biological functions of PtWRKYs were analyzed, and it was found that PtWRKYs had potential nitrogen response functions (Figure 5). The regulatory network of PtWRKY TFs on nitrogen-responsive genes is predicted, and it is suggested that PtWRKYs may directly or indirectly regulate the expression levels of many nitrogen-responsive genes, including *NRTs, ASN, NR, NiR* and so on (Figure 6). In addition, bioinformatic analysis confirmed that the nitrogen responsive PtWRKYs were involved in the response of ABA and other plant hormone responses (Figure 4). Thus, we speculated that these PtWPKYs might be involved in the regulation of crosstalk between nitrogen and multiple other signals, and even presumably become the key node by which NO_3_^-^ signaling causes the system response of poplar. We should pay great attention to the crosstalk effect of PtWRKYs on plant hormone signals and the systemic regulatory mechanism of PtWRKYs on poplar adaptability under fluctuating nitrogen conditions. Further analysis and experiments to confirm this process will be very meaningful.

## 5. Conclusions

In this study, 98 *PtWRKYs* were identified by an HMM search and further classified into seven subgroups, namely, I, II (IIa, IIb, IIc, IId, IIe), and III, which included 21, 5, 9, 24, 13, 16 and 10 *PtWRKYs*, respectively. Two genes incorrectly annotated as WRKY were corrected. Phylogenetic analysis and the promoter cis-acting element detection revealed that PtWRKYs have functions in stress response, morphogenesis and nutrition utilization. As shown in ML-hGRNs, many WRKY TFs were predicted to be involved in the nitrogen response, such as PtWRKY33, PtWRKY95, and PtWRKY40. They mainly regulated the expression of primary NRGs to influence nitrogen transport and assimilation. Expression profile analysis showed that 6 and 15 *PtWRKYs* were regulated by available nitrogen content in leaves and roots, respectively, and most of them were predicted in ML-hGRN. In this study, the structural and functional characteristics of the WRKY gene family in poplar were analyzed in detail, and PtWRKYs were found to be involved in the nitrogen response in poplar. This study lays a solid foundation for further functional analysis of the WRKY gene family and breeding improvement for nitrogen use efficiency and wood performance in poplar.

## Data Availability

Not applicable.

## Supplementary Materials

The following supporting information can be downloaded at: https://www.mdpi.com/article/10.3390/genes13122324/s1, Figure S1: Alignment of WRKY domain in *P. trichocarpa.* Table S1: Gene expression matrixes of TFs and NRGs downloaded at Phytozome used in ML-hGRN construction. Table S2: Name and physicochemical property of poplar WRKY TFs identified by hmmsearch. Table S3: Functional annotations of WRKY TFs based on previous studies.
