# Supplementary figures and images for: Genome-Wide Identification of WRKY Family Genes and the Expression Profiles in Response to Nitrogen Deficiency in Poplar
Source: Genes (Basel). 2022 Dec 10;13(12):2324. doi: 10.3390/genes13122324 (PMC9777946; doi:10.3390/genes13122324)

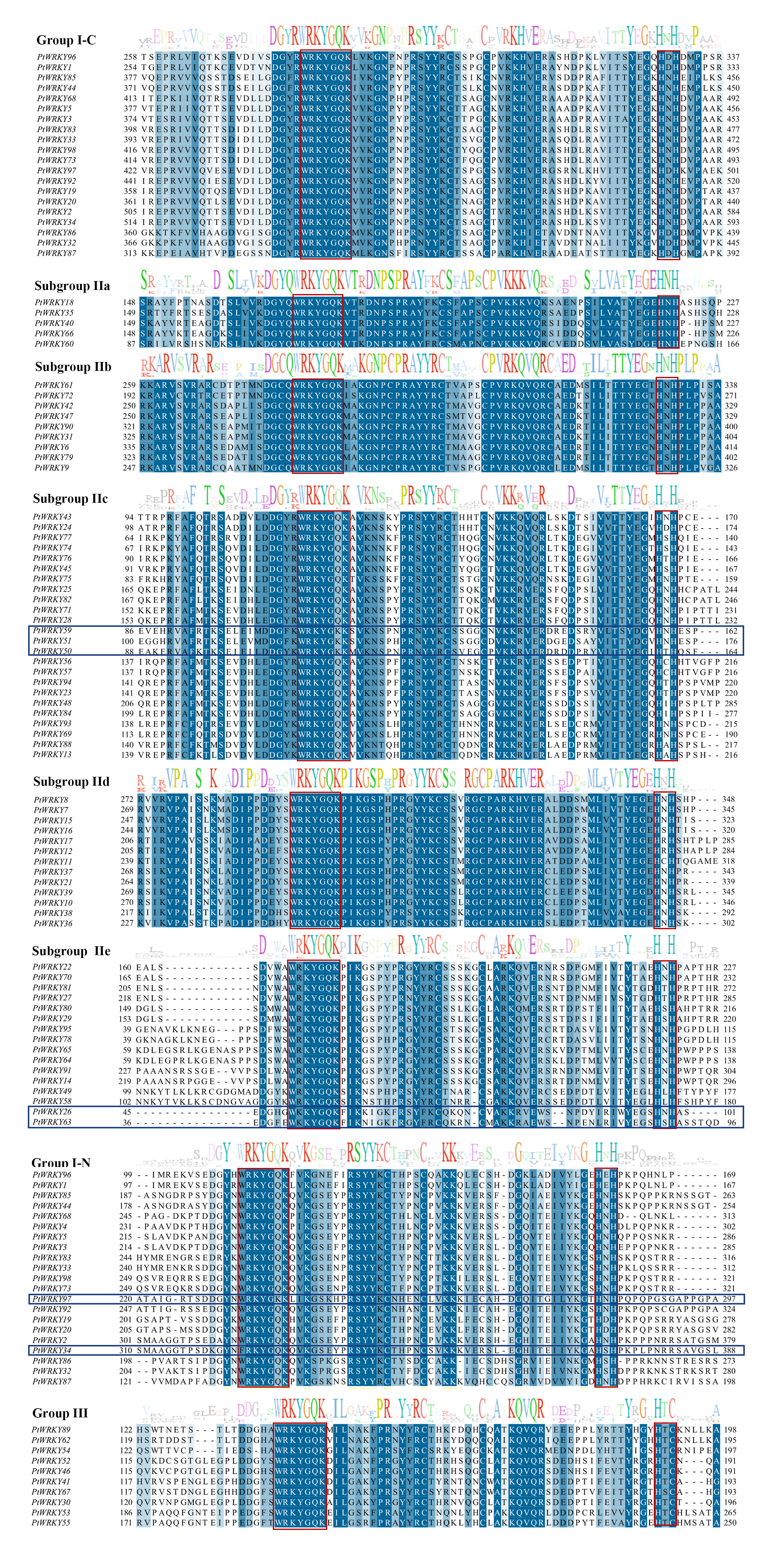

Supplement: Supplementary file 1 [file genes-13-02324-s001.zip › Figure S1.png]
